# Safety of SARS-CoV2 vaccination and COVID-19 short-term outcome in pediatric acquired demyelinating disorders of central nervous system: A single center experience

**DOI:** 10.3389/fimmu.2023.1106472

**Published:** 2023-01-25

**Authors:** Gabriele Monte, Laura Papetti, Michela Ada Noris Ferilli, Fabiana Ursitti, Romina Moavero, Giorgia Sforza, Elena Panella, Samuela Tarantino, Martina Proietti Checchi, Federico Vigevano, Paolo Palma, Massimiliano Valeriani

**Affiliations:** ^1^ Developmental Neurology Unit, Bambino Gesù Children’s Hospital, Istituto di Ricovero e Cura a Carattere Scientifico (IRCCS), Rome, Italy; ^2^ Child Neurology and Psychiatry Unit, Department of System Medicine, Tor Vergata University of Rome, Rome, Italy; ^3^ Clinical Immunology and Vaccinology Unit, Bambino Gesù Children’s Hospital, Istituto di Ricovero e Cura a Carattere Scientifico (IRCCS), Rome, Italy; ^4^ Chair of Pediatrics, Department of Systems Medicine, Tor Vergata University of Rome, Rome, Italy; ^5^ Center for Sensory-Motor Interaction, Aalborg University, Denmark, Neurology Unit, Aalborg, Denmark

**Keywords:** SARS-CoV2, COVID-19 vaccination, safety, multiple sclerosis, MOGAD, NMOSD

## Abstract

**Introduction:**

Concern of a correlation between disease relapse in patients with acquired demyelinating disorders of central nervous system (CNS) and SARS-CoV2 vaccines has been raised. In this single center study, we retrospectively evaluated safety of SARS-CoV2 vaccination and COVID-19 short-term outcome in pediatric acquired demyelinating disorders of CNS.

**Materials and methods:**

Patients with multiple sclerosis (MS), myelin oligodendrocyte glycoprotein antibody associated disease (MOGAD) and neuromyelitis optica spectrum disorder (NMOSD) with disease onset before 18 years of age were included. Demographic and clinical data, and information regarding previous SARS-CoV-2 infection and vaccination were collected.

**Results:**

We included nine patients with MOGAD. Six patients received SARS-CoV2 vaccination and complained pain at injection site while only one had fever and fatigue. Median follow-up was 28 weeks (range 20-48). Seven patients had COVID-19 occurring with mild flu-like symptoms and median follow-up was 28 weeks (range 24-34). Nobody had disease relapse. Five patients with NMOSD were included. All patients received SARS-CoV2 vaccination (BNT162b2-Pfizer-BioNTech). The median follow-up was 20 weeks (range 14-24) and only two patients complained pain at injection site, fever and fatigue. Three patients had also COVID-19 with mild flu-like symptoms, despite two of them being under immunosuppressive treatment. Lastly, forty-three patients with MS were included. 35 out of 43 received SARS-CoV2 vaccination with a median follow-up of 24 weeks (range 8-36). Fourteen patients had no side effects, while 21 complained mild side effects (mainly pain at injection site) and one experienced a disease relapse with complete recovery after steroid therapy. At vaccination, all but one were under treatment. Sixteen patients had COVID-19 occurring with mild symptoms.

**Discussion:**

COVID-19 outcome was good although many patients were under immunosuppressive treatment. Vaccine-related side effects were frequent but were mild and self-limited. Only one MS patient had a post-vaccination relapse with complete recovery after steroid therapy. In conclusion, our data support the safety of SARS-CoV-2 vaccines in pediatric MS, MOGAD and NMOSD.

## Introduction

Coronavirus disease 2019 (COVID-19) pandemic, caused by severe acute respiratory syndrome coronavirus 2 (SARS-CoV2), had a devastating impact on public health. In response, there has been development and deployment of vaccines at an unprecedented speed and scale (1). Since vaccination acts through a strong induction of the immune system, a concern of a correlation between disease relapse in patients with acquired demyelinating disorders of central nervous system (CNS) and SARS-CoV2 vaccines has been raised (2). In the past two decades, several studies have shown the absence of a causal link between vaccination and multiple sclerosis (MS) relapse (3). Moreover, the safety of SARS-CoV2 vaccines has been highlighted in adult MS (4) and in adult patients with aquaporin-4-IgG neuromyelitis optica spectrum disorder (AQP4-IgG+NMOSD) and myelin oligodendrocyte glycoprotein associated disease (MOGAD) (5). There is a substantially higher risk of demyelination following SARS-CoV2 infections compared to vaccination (6) and disease relapses with superimposed infection may cause more severe and sustained disability than spontaneous ones (7). Indeed, several studies suggest that it is of utmost importance for people with NMOSD and MS to minimize the risk of SARS-CoV2 infection, considering that infection-associated adverse events outweigh the risks of vaccination (8, 9).

BNT162b2-Pfizer-BioNTech and mRNA-1273-Moderna have gained approval for the pediatric age but there are limited data on the safety in pediatric acquired demyelinating disorders of CNS. The lack of data might contribute to parents’ hesitancy toward childhood SARS-CoV2 vaccination, that is one of the top public health threats. Reassuring hesitant parents on the safety of vaccine is essential. Therefore, we evaluated safety and risk of disease relapse after vaccination in our cohort of pediatric acquired demyelinating disorders of CNS and we also investigated COVID-19 outcome.

## Materials and methods

We retrospectively included patients with MS, MOGAD and NMOSD with disease onset before 18 years of age. Demographics, clinical data and information regarding previous SARS-CoV2 infection and vaccination were collected. Disease relapses were considered “post-infectious” or “post-vaccination” when occurring within 4 weeks from infection or vaccination. Continuous and categorical variables were reported as median (range) and proportion (percentage), respectively. Consent for the use of medical data for research purposes was obtained. The study was approved by the Institutional Review Board of our institution.

## Results

We included nine patients with MOGAD. Six patients received SARS-CoV2 vaccination with a median age at first dose of 11 years (range 6-15): five received BNT162b2-Pfizer-BioNTech and one mRNA-1273-Moderna. Patients had a monophasic disease and the median disease duration at first dose was 35 months (range 14-53).All complained pain at injection site and one had also fever and fatigue. Two patients received the second dose of BNT162b2-Pfizer-BioNTech reporting pain at injection site. Follow-up after the last vaccination was 28 weeks (range 20-48). Nobody was under immunosuppressive treatment at vaccination and four patients had already had SARS-CoV2 infection. Seven patients had COVID-19 occurring with mild flu-like symptoms with a median disease duration of ten days (range 7-14). Two of them had already received two doses of vaccine. Post-infection follow-up was 28 weeks (range 24-34). No patient had disease relapse, neither after vaccination nor after infection.

Five patients with NMOSD were included, three AQP4-IgG+ and two seronegative. At first vaccination the median age was 16 years (range 10-19), the median disease duration was 40 months (range 24-110) and the time from last relapse was 36 months (range 13-70).All patients received two doses of BNT162b2-Pfizer-BioNTech and only two (40%) complained pain at injection site, fever and fatigue. Median follow-up after the last dose was 20 weeks (range 14-24) and nobody had disease relapse. After vaccination, three patients had also COVID-19 occurring with mild flu-like symptoms, despite two of them being under immunosuppressive treatment (one was treated with rituximab and the other with mycophenolate mofetil). At vaccination, three patients were under 18 years of age and only one complained pain at injection site, fever and fatigue with a median follow-up after the last dose of 16 weeks (range 20-24). Forty-three patients with MS were included with a median age at first dose of 16 years (range 10-22). At first vaccination the median disease duration was 16 months (range 4-78) and the time from last relapse was 13 months (range 3-60). Thirty-five out of 43 received SARS-CoV2 vaccination: 31 received BNT162b2-Pfizer-BioNTech and 4 mRNA-1273-Moderna. At least two doses were administered and fourteen patients (40%) reported no side effects. At vaccination, all but one were under treatment: six with fingolimod, sixteen with natalizumab, three with anti-CD20 therapy, three with dimethyl fumarate, four with interferon beta and two with glatiramer acetate. The median follow-up after the last vaccination was 24 weeks (range 8-36) and only one patient experienced a disease relapse two weeks after the first dose of BNT162b2-Pfizer-BioNTech with complete recovery after steroid therapy. After the first and second dose, twenty-one patients (60%) complained pain at injection site and five of them had also headache, fatigue and fever. Sixteen patients received the third dose and ten reported pain at injection site, of which three had also fever and fatigue. Considering only patients under the age of 18 at vaccination, we included twenty-eight patients with MS. After the first and second dose, seventeen patients (61%) complained pain at injection site and three of them also reported headache, fever and fatigue with a follow-up of at least 8 weeks. Nine patients received the third dose and six reported pain at injection site, of whom two had also fever and fatigue. The patient who experienced the disease relapse was in this age subgroup.

Eight patients had COVID-19 after vaccination with mild flu-like symptoms and a median disease duration of 8.5 days (range 5-14). Non-vaccinated patients had COVID-19 occurring with mild flu-like symptoms, but they had a longer disease duration (median 10 days, range 7-20). Sixteen patients, who were under immunosuppressive treatment while experiencing COVID-19, had no relapse.

In our cohort, there were not events of anaphylaxis or life-threatening responses after vaccination. Clinical characteristics and data related to SARS-CoV2 infection and vaccination are summarized in **Table 1**.

**Table 1:** Clinical characteristics, SARS-CoV2 vaccine safety profile and COVID-19 outcome of included patients.

	MOGAD (n=9)	NMOSD (n=5)	MS (n=43)
Female, n (%)	4 (45%)	3 (60%)	26 (60%)
Vaccinated patients, n (%)	6 (67%)	5 (100%)	35 (81%)
Median age at first dose, years (range)	11 (6-15)	16 (10-19)	16 (10-22)
Total number of doses received per patient	3 doses: 0 2 doses: 2 1 dose: 4	3 doses: 0 2 doses: 5 1 dose: 0	3 doses: 16 2 doses: 19 1 dose: 0
Type of vaccine received at first dose	Pfizer 5 Moderna 1	Pfizer 5 Moderna 0	Pfizer 31 Moderna 4
Type of vaccine received at second dose	Pfizer 2 Moderna 0	Pfizer 5 Moderna 0	Pfizer 31 Moderna 4
Type of vaccine received at third dose	Pfizer 0 Moderna 0	Pfizer 0 Moderna 0	Pfizer 14 Moderna 2
Median follow-up after last dose, weeks (range)	28 (20-48)	20 (14-24)	24 (8-36)
DMT at vaccination	No treatment 6 (100%)	MMF 1 RTX 2 IVIG 1 No treatment 1	Fingolimod 6 Natalizumab 16 RTX 3 Dimethyl fumarate 3 Interferon beta 4 Glatiramer acetate 2 No treatment 1
Disease relapse after vaccination, n (%)	0 (0%)	0 (0%)	1 (2.9%)
Side effects after the first dose	Local pain 6 (100%) Fever 1 (17%) Fatigue 1 (17%) Headache 0	Local pain 2 (40%) Fever 2 (40%) Fatigue 2 (40%) Headache 0	Local pain 21 (60%) Fever 5 (14%) Fatigue 5 (14%) Headache 5 (14%)
Side effects after the second dose	Local pain 2 (100%) Fever 0 Fatigue 0 Headache 0	Local pain 2 (40%) Fever 2 (40%) Fatigue 2 (40%) Headache 0	Local pain 21 (60%) Fever 5 (14%) Fatigue 5 (14%) Headache 5 (14%)
History of COVID-19	7 (78%)	3 (60%)	16 (37%)
COVID-19 symptoms	Mild flu-like symptoms	Mild flu-like symptoms	Mild flu-like symptoms
Disease relapse after COVID-19	0	0	0

DMT, disease modifying treatment; IVIG, intravenous immunoglobulins; Moderna, mRNA-1273-Moderna; MMF, mycophenolate mofetil; MOGAD, myelin oligodendrocyte glycoprotein antibody associated disease; MS, multiple sclerosis; NMOSD, neuromyelitis optica spectrum disorder; Pfizer: BNT162b2-Pfizer-BioNTech; RTX: rituximab.

## Discussion

In this study, we evaluated whether SARS-CoV2 vaccination is associated with adverse events and disease relapse in pediatric acquired demyelinating disorders of CNS. Our patients reported mild and self-limited adverse events (i.e. pain at injection site, headache, fever and fatigue) as observed in the general population, even when we considered only patients under 18 years of age at vaccination (10). Frequency of post-vaccination disease relapses was 0% in MOGAD and NMOSD, consistent with other studies (5, 11, 12). One MS patient experienced a post-vaccination relapse, meaning that 2.9% of our MS patients showed a relapse after SARS-CoV2 vaccination. This value is in line with another study that also found a similar relapsing rate in non-vaccinated patients during the same time interval (4). The patient who had a relapse fully recovered without long-term sequelae after steroid therapy, as already observed in most patients with post-vaccination neurological complications (6). COVID-19 outcome was good, despite most of our patients were on immunosuppressive therapy. This finding could be related to the absence of comorbidities, such as hypertension and diabetes, which are important risk factors for severe COVID-19 in MS and NMOSD (8, 13). In conclusion, our data support the safety and tolerability of SARS-CoV2 vaccines in pediatric MS, MOGAD and NMOSD, which should be strongly recommended. This study has some limitations, such as the small number of patients included from a single center, the retrospective design and exclusive exposure to mRNA-based vaccines. Further studies are needed to confirm these findings and to counteract vaccine hesitancy.

## Data availability statement

The raw data supporting the conclusions of this article will be made available by the authors, without undue reservation.

## Ethics statement

The studies involving human participants were reviewed and approved by Bambino Gesù Children’s Hospital. Written informed consent to participate in this study was provided by the participants’ legal guardian/next of kin.

## Author contributions

GM, LP, MF, FU, RM, GS, EP, ST, and MP acquired the clinical data. GM and MV drafted the manuscript. GM, MV, PP and FV critically revised the manuscript. All authors contributed to the article and approved the submitted version.
